# Epithelial Fluid Transport is Due to Electro-osmosis (80%), Plus Osmosis (20%)

**DOI:** 10.1007/s00232-017-9966-x

**Published:** 2017-06-16

**Authors:** Jorge Fischbarg, Julio A. Hernandez, Andrey A. Rubashkin, Pavel Iserovich, Veronica I. Cacace, Carlos F. Kusnier

**Affiliations:** 1 Ininca, Conicet, Univ. of Buenos Aires2, Buenos Aires, Argentina; 2 Biophysics Section, Science Faculty, Univ. of the Republic, Montevideo, Uruguay; 3 Institute of Cytology of the Russian Academy of Science, St. Petersburg, Russia; 4 SUNY Downstate, New York, USA

**Keywords:** Fluid transport, Electro-osmosis, Osmosis

## Abstract

Epithelial fluid transport, an important physiological process shrouded in a long-standing enigma, may finally be moving closer to a solution. We propose that, for the corneal endothelium, relative proportions for the driving forces for fluid transport are 80% of paracellular electro-osmosis, and 20% classical transcellular osmosis. These operate in a cyclical process with a period of 9.2 s, which is dictated by the decrease and exhaustion of cellular Na^+^. Paracellular electro-osmosis is sketched here, and partially discussed as much as the subject still allows; transcellular osmosis is presented at length.

## Historical Perspective of the Question

We take the corneal endothelium as an example for fluid transporting epithelia. In this mono-cellular layer, two different mechanisms of fluid transport appear reasonably separated: (1) paracellular electro-osmosis (80% of the total) and (2) transcellular osmosis (20%). Of course, there is no limit for how complicated can the matter ultimately grow in more anatomically complex layers. We will end up with a simple blueprint whose elements can be superimposed on any anatomy. The tale can be summarized graphically in a few diagrams depicting the histology of the endothelium and the surrounding layers, and the main physiological events resulting in fluid transport.

**Fig. 1:**
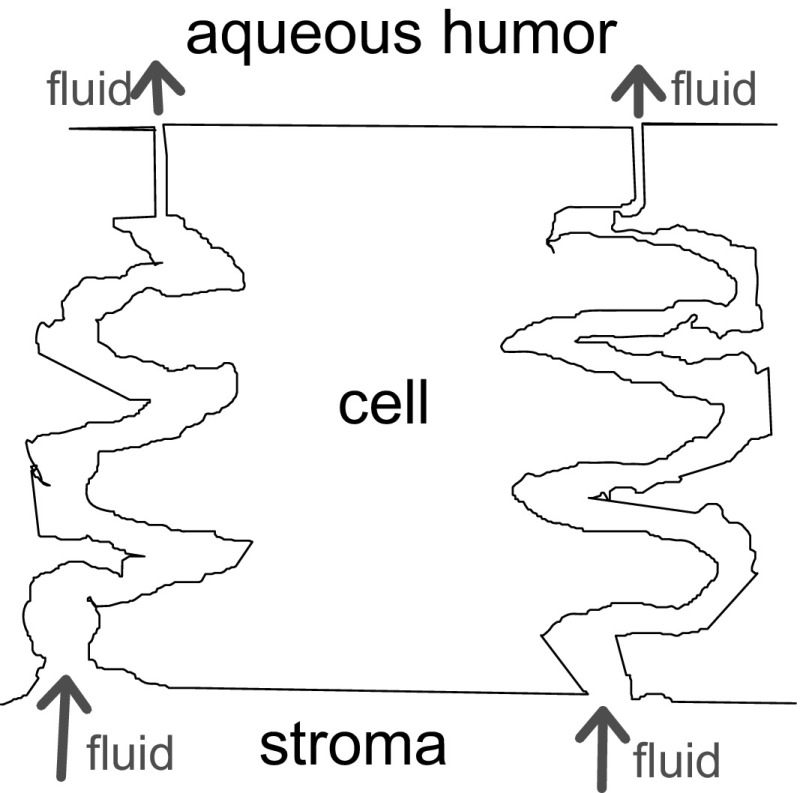
Corneal endothelial cell, surrounded by its limiting layers and by a very convoluted intercellular space. The stroma is anterior, the aqueous is posterior. The direction of fluid transport, forced through a bottleneck, is highlighted

**Fig. 2:**
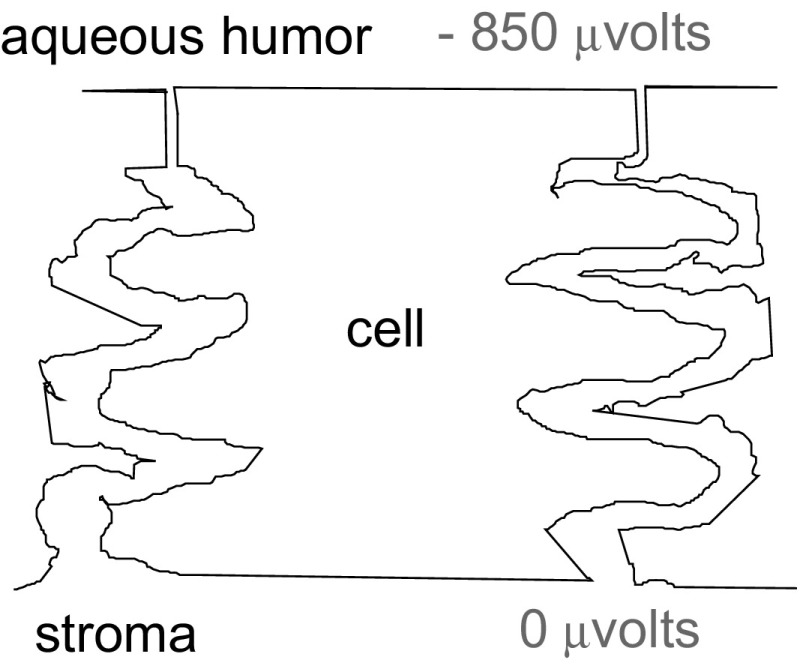
There is a standing electric field along the endothelium. Stroma is anterior; aqueous is posterior

So we begin with Fig. 1, which presents an overall display of the “battlefield.” Dimensions given are for the rabbit corneal endothelium, which we take as our standard preparation on the basis of two coincident morphological reports (Oh 1970; Sailstad and Pfeiffer 1981). The cells (~4 μm tall) lie sandwiched between the corneal stroma (in the anterior, or blood-side direction), and the aqueous humor (in the posterior, or body-cavity direction, this body cavity is also called the “anterior chamber”). The *intercellular space* between endothelial cells is one of the distinctive keys of the present story. It is shown here (Fig. 2) convoluted in an idealized diagram. In the rabbit, if straightened out, it would measure in length 12 μm from end to end. Its width is about 20 nm, but in its final distal 1 µm it narrows down considerably to a width of only 42.5 Å or 4.25 nm. This narrow end abuts into the anterior chamber, constitutes the “tight junction,” and results in a bottleneck for any flows of matter across the paracellular pathway between the stroma and the aqueous.

Such bottleneck is crucial. It so happens that endothelial fluid transport goes in a particular direction, from stroma towards aqueous, that is to say, through the high resistance bottleneck. This was shown by three different laboratories in a remarkably coincident spat of research (Fischbarg 1972; Dikstein and Maurice 1972; Hodson 1974). Any hypothetical osmotic, diffusional, or hydrostatic temporal buildup of fluid inside the lateral spaces, if left to its own resources, would flow out naturally in the direction of least resistance, that is, in the anterior direction towards the wide open stromal end. Active transport of fluid however goes in the exact *opposite* direction, that is, from stroma towards aqueous. We discard peristaltic motions of the intercellular spaces because of lack of evidence. Hence, the conclusion is forced: the only physical process that can possibly account for such evidence is paracellular electro-osmosis. It is fitting that experimental evidence supports this view (Sanchez et al. 2002, 2016).

Given that this electro-osmotic transfer of fluid is occurring, where does it originate? We would think there is an intense electric field along the paracellular junction, aqueous being negative. Of course, the field is there: 850 μV μm^−1^ in the rabbit (Fig. 2) (Fischbarg 1972). How it originates, it is still controversial; we have argued for an electrogenic apical Na^+^/3${\text{HCO}}_{3}^{ - }$ cotransporter (Diecke et al. 2004), others debate that (Bonanno 2012). Whatever the explanation, we will forge ahead noting the experimental finding that such a large electric field does exist across the endothelium (Fischbarg 1972; Barfort and Maurice 1974; Hodson 1974).

**Fig. 3:**
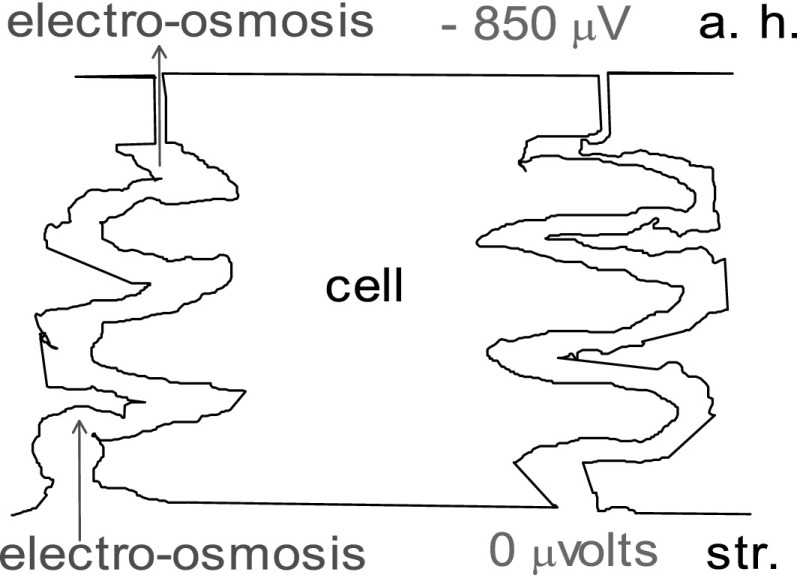
The electrical gradient generates electro-osmosis

Next in line would come a hypothetical mass of cations, freshly secreted into the intercellular space, ready to carry the electro-osmosis current (Fig. 3). There is separate evidence for this as well: a large density of Na^+^ pumps have been found in the lateral wall of the endothelial cells (Geroski and Edelhauser 1984), all along the paracellular space. The authors found a density of 3 × 10^6^ pump sites cell^−1^.

Still one more element is required at this point. For electro-osmosis to occur most efficiently, it would be required that the intercellular junction would be very selective towards positive ions, and would reject the vast majority if not all of Cl^−^ ions. There is evidence for junctions being selective towards positive ions (Lim et al. 1983). In addition, junctions have special properties (Fukushima et al. 2015) derived from molecular crowding in their narrow space, so it is conceivable that the effective exclusion of anions could be larger than expected.

Taking all together, we come up with an intense ionic current through the junctions, generating electro-osmotic coupling (80%) with the fluid. The end result is a large mass of fluid from the stroma and lateral space being transferred to the apical space. In addition, separately, a small component of classical osmosis (20%) develops in the same direction.

### Cyclic Behavior: (1) Our Model for Osmotic and Non-osmotic Transports

The process of sodium-dependent electro-osmotic flow across the intercellular junction cannot go on continuously, because the supply of cell Na^+^ ions is small, and transport into the lateral space tends to exhaust such supply rapidly. How long would it take for the cell to run out of Na^+^ ions? For the rabbit, the net flux of Na^+^ (from stroma to aqueous) is (Lim and Ussing 1982)

$${\text{Na}}\_{\text{f}} = 2.3 \times 10^{ - 6} \;{\text{mole}}\;{\text{h}}^{ - 1} \;{\text{cm}}^{ - 2} .$$

We assume such flux all goes through the junctions. Their cross-sectional area is much smaller (4.33 × 10^−4^ cm^2^ cm^−2^ of tissue). Hence, the junctional Na^+^ flux becomes:

$${\text{Na}}\_{\text{f}}\_{\text{j}} = 5.3 \times 10^{ - 3} \;{\text{mole}}\;{\text{h}}^{ - 1} \;{\text{cm}}^{ - 2} .$$

Now we focus on the Na^+^ flux through one *segment of half junction*, through which it exits the Na^+^ that originates from a single side of the cell, corresponding to that junction. The area of such segment of half junction is:

$${\text{Ash}}_{\text{j}} = {\text{half}}\;{\text{junctional}}\;{\text{width}} \times {\text{length}}\;{\text{of}}\;{\text{one}}\;{\text{side}}\;{\text{of}}\;{\text{the}}\;{\text{hexagonal}}\;{\text{cell}} = 21.25\;{\text{\AA}} \times 11.3\;\upmu {\text{m}} = 0.024\;\upmu {\text{m}}^{2} .$$

The corresponding area of the segment of cell from which the exiting Na^+^ flux originates is:

$${\text{As}}_{\text{c}} = {\text{height}}\;{\text{cell}}\;{\text{membrane}}\;(12\;\upmu {\text{m)}} \times {\text{length}}\;{\text{one}}\;{\text{hex}}\;{\text{side}}\;{\text{cell}}\;(11.3\;\upmu {\text{m)}} = 135.9\;\upmu {\text{m}}^{2} .$$

Now, the ratio of the two areas (segment of half junction over segment of cell) is:

$$r_{\text{A}} = {\text{Ash}}_{\text{j}} /{\text{As}}_{\text{c}} = 1.77 \times 10^{ - 4} .$$

This yields the Na^+^ flux through one segment of the lateral cell membrane. It is:

$$F_{\text{cm}} = {\text{Na\_f}}\_{\text{j}} \times r_{\text{A}} = 9.4 \times 10^{ - 7} \;{\text{mole}}\;{\text{h}}^{ - 1} \;{\text{cm}}^{ - 2} .$$

The Na^+^ flux through the total lateral cell membrane area is simply six times that times the lateral area of one cell segment, or:

$${\text{TF}}_{\text{cm}} = F_{\text{cm}} \times 6 \times {\text{As}}_{\text{c}} = 2.13 \times 10^{ - 3} \;{\text{pmole}}\;{\text{s}}^{ - 1} .$$

### Cyclic Behavior: (2) Electro-osmosis: The Water and Ionic Cycles

The volume of one endothelial cell is 1.333 × 10^3^ µm^3^ (cf. refs. above). At 14.6 mM Na^+^ intracellular concentration, the Na^+^ contents of one cell are 19.4 fmole. That means that a cell would empty out of Na^+^ in 9.1 s. This is a key result. It begins to explain why half a cycle lasts only 4.8 s, while a cell unloads parts of its Na^+^, and why the following replenishment half cycle lasts the same time, while a cell replenishes its Na^+^. When after a few seconds the original ionic gradients are reconstituted, the process leading to electro-osmosis can start over. Such cyclic behavior was indeed found a few years ago (Montalbetti and Fischbarg 2009) and it is only now being fully explained (Cacace et al. 2011). The complete cycle is 9.2 s.

**Fig. 4:**
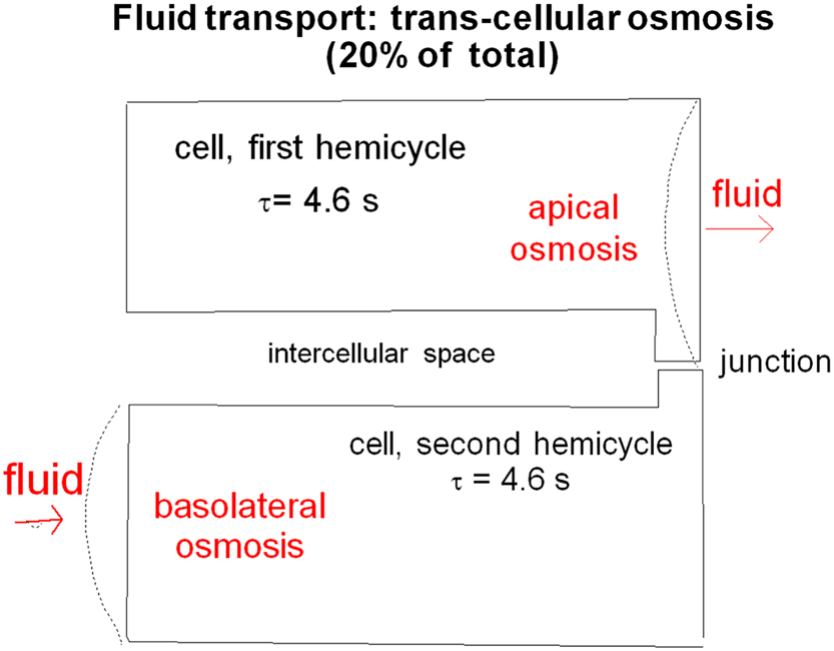
Transcellular classical osmosis occurs as well. During the first half cycle, water exits the cell through the apical membrane, and during the second half cycle, water enters the cell through the basolateral membrane

During the first half of the cycle, paracellular electro-osmosis is in full development. Na^+^ flows in a loop, exiting the cell body via the intercellular space, and re-entering the cell via the apical Na^+^ channels. The junctional Na^+^ movement drags water along (electro-osmosis) from apex to base, forming the bulk of the fluid secretion. Some water may be transferred by osmosis (from cell to aqueous, Fig. 4). This resembles the volume loss that occurs during regulatory volume decrease. The excess apical Na^+^ that drives electro/osmosis is subsequently reabsorbed by the cell through apical Na^+^ channels. In fact, it has been noted that epithelial Na plasma membrane channels appear predominantly in the membrane neighborhood closest to the junctions, as if to functionally minimize the travel time needed.

### Cyclic Behavior: (3) Osmosis

Subsequently, at the end of the second half time of the cycle, the apical transjunctional Na^+^ transport and the resulting water electro-osmotic movement cease. This can be marked as zero time, the beginning of a cycle. At this point, the cell then begins to regain lost electrolytes mainly via the basal Na/K/2Cl cotransporter (Fischbarg 1999). This is accompanied by the entry into the cell of a small volume of fluid (Fig. 4), this time across its basal membrane (Kuang et al. 2001), in a process that very much resembles the volume gain of regulatory volume increase. All sides of the cell are rich in aquaporin 1 in its plasma membranes (Li et al. 1999), which of course makes these rapid 5 s compensatory osmotic shifts in volume, possible (Li et al. 1999).

### Cyclic Behavior Summarized

In summary, the phase of cycles includes the following: (1) commencement via electro-osmotic secretion (from base to apex) of Na^+^ and an accompanying paracellular water column (80% of the total volume). (2) Subsequent fine adjustment via salt and water basal uptake (20% of total transported water).

### General Comments

A further word on these osmotic flows appears in order. Given that they are 20% of the total, could they possibly grow to be substantially larger? The answer is: most definitely no. Osmotic flows now here near enough for the task. We limit our analysis to the apical membrane, the area of which is smaller than that of the basolateral one. Given its osmotic permeability of 78 µm s^−1^ (Echevarria et al. 1993), a steady state fluid transport flow of the observed magnitude of 45 µm h^−1^ would require a concentration gradient at the apical membrane of 9 mM l^−1^ of salt. But that requirement carries implicit a fantasy. The unstirred layer there is between 60 and 350 µm (Green and Otori 1970), which means such gradient would result in a standing net outward flow of salt of between 65 and 11 mM of salt h^1^ cm^2^. However, biologically observed flows are very far from such large numbers, only 2–4 in such units. A similar rebuttal has been already published (Fischbarg 2010).

There are a number of issues that remain. K^+^ and Cl^−^ channels (and transporters) could have a role; the apical electrogenic Na^+^/${\text{HCO}}_{3}^{ - }$ cotransporter would have to be inactivated during the second hemicycle; the overall cell signaling process is incompletely known; and so on. However it seems that, in the fundamental, we have a cogent basic sequence on which to base future analysis, for this and many other similar epithelial layers.

## Mathematical Model of the Cyclic Mechanism

**Fig. 5:**
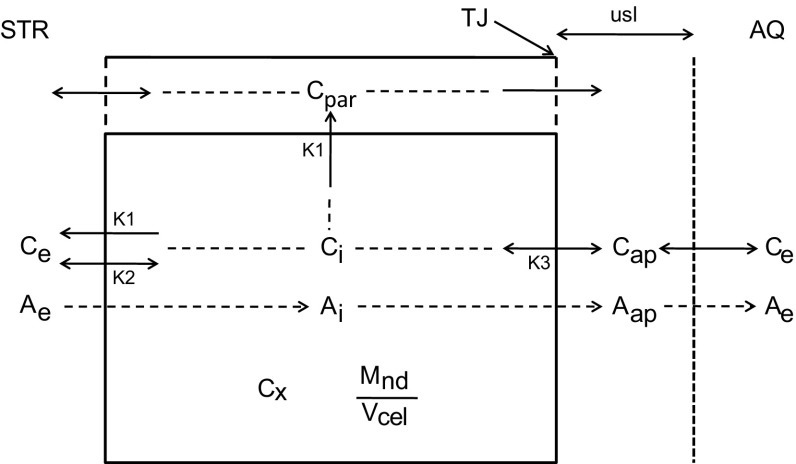
Scheme of an epithelial cell utilized to derive the mathematical model

**Fig. 6:**
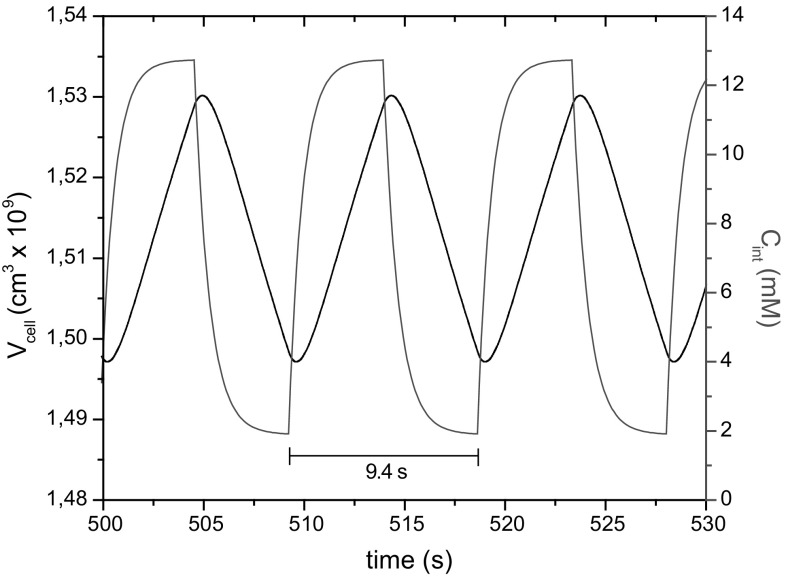
Plots of *V*_cell_ and *C*_int_ versus time obtained from the numerical integration of the model derived in the Appendix

**Fig. 7:**
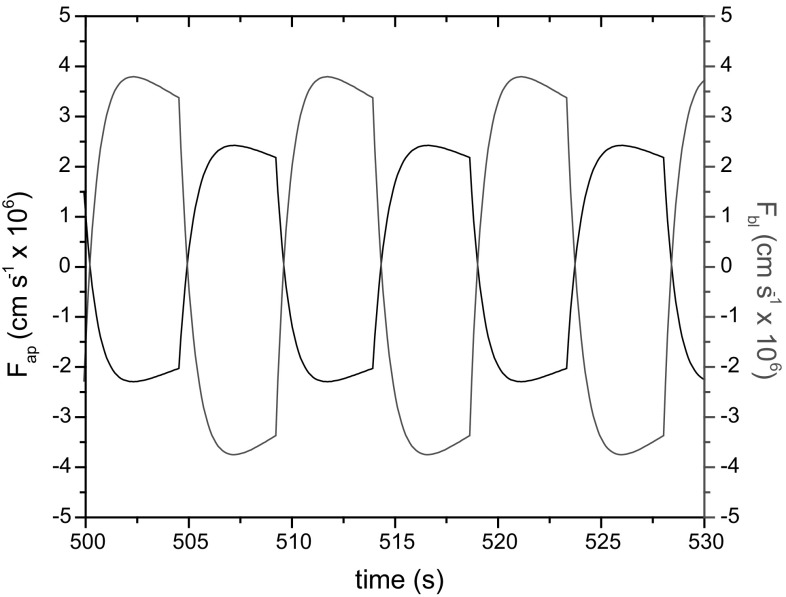
Plots of *F*_ap_ and *F*_bl_ versus time obtained from the numerical integration of the model derived in the Appendix

**Fig. 8:**
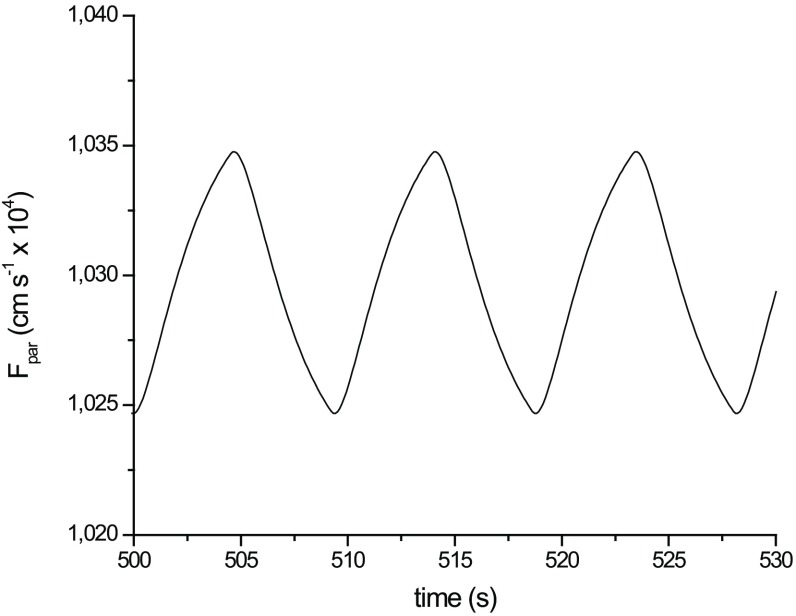
Plot of *F*_par_ versus time obtained from the numerical integration of the model derived in the Appendix

We developed a schematic model to test the plausibility of the proposed mechanism (Appendix). The model considered dimensional data characteristic of rabbit corneal endothelial cells and kinetic parameters were chosen to account for experimental results on sodium and fluid transport across this tissue. The mechanism assumed active transport of a cation C (e.g., sodium) from the cell to the intercellular space, electrodiffusion of C driven by the transepithelial electrical potential difference, and accumulation and cell re-entry of C at the apical membrane (Fig. 5). To account for the oscillatory behavior, we incorporated short-term cell exhaustion of the contents of C. The numerical simulations of the model reveal a periodic operational regime, characterized by oscillations in the cell volume, the cell osmolarity and the water flows, with a time period of 9.4 s (Figs. 6, 7, 8). For the parameter values utilized, both the electro-osmotic and the net osmotic water flows were positive in the basal (stromal, STR) to apical (aqueous, AQ) direction, with values of 3.73 × 10^−3^ and 1.07 × 10^−3^ cm h^−1^, respectively. The total water flow obtained by the numerical integration of the model, 4.8 × 10^−3^ cm h^−1^, was thus similar to the experimental one (4.3 ± 0.6 × 10^−3^ cm h^−1^) (Narula et al. 1992). Analogously, sodium flux occurred in the basal to apical direction with a value of 1.8 × 10^−6^ mole cm^−2^ h^−1^, not far from experimental data (2.3 ± 0.4 × 10^−6^ mole cm^−2^ h^−1^) (Lim and Ussing 1982). We believe that the behavior and the numerical results of the model simulations support the plausibility of the mixed mechanism proposed to explain net fluid movement coupled to solute transport in epithelia, particularly considering that a schematic representation is already capable to provide good approximations to the available experimental evidence.

## Appendix

### A Dynamic Model of Oscillatory Fluid Transport Across Epithelia

Figure 5 shows the scheme of an epithelial cell utilized to derive the model. It is assumed that a monovalent cation C and an accompanying monovalent anion A are transported from the basal (stromal, STR) to the apical (aqueous, AQ) side. Three main compartments are considered: intracellular, paracellular, and an unstirred layer at the apical side (usl). The concentrations of C in these compartments (*C*_i_, *C*_par_, and *C*_ap_, respectively) vary during the transport cycle. We assumed that, due to electroneutrality, the concentration of A in each one of the compartments always remains equal to that of C. Across the plasma membrane domains, C is subject to passive (double arrows) and active (single arrow) transports, governed by rate constants *K*_1_ (active) and *K*_2_ and *K*_3_ (passive). At the paracellular way, C enters via a reversible diffusive mechanism (double arrow) and exits at the tight junction (TJ) due to electrodiffusion determined by the transepithelial potential difference (single arrow).

### Model Parameters

- *V*_w_: partial molar volume of water,
- *S*_e_: total extracellular solute concentration,
- *C*_e_, *A*_e_: extracellular concentrations of C and A (*C*_e_ = *A*_e_),
- *A*_c_: contact area between the cell and the extracellular compartment at the apical and basal domains (per cell),
- *A*_l_: contact area between the cell and the paracellular space (per cell),
- *A*_tj_: contact area between the paracellular space and the apical usl (i.e., at the level of the TJ, per cell),
- *A*_par_: contact area between the paracellular space and the extracellular compartment at the apical site (per cell),
- *R*_act_: density fraction of C pumps at the basal domain,
- *V*_ap_: volume of the usl at the apical side (per cell),
- *V*_par_: volume of the paracellular space (per cell),
- *M*_nd_: total intracellular mass of non-diffusible solutes (per cell),
- *C*_*x*_: total concentration of intracellular diffusible solutes different from A and C,
- *K*_1_: rate constant of active transport of C,
- *K*_3_(α), *K*_2_(α): rate constants of passive transport of C for the steady state α,
- *K*_3_(β), *K*_2_(β): rate constants of passive transport of C for the steady state β,
- *K*_d_: rate constant of transport of C between the usl and the extracellular medium,
- *K*_4_: rate constant of transport of C between the extracellular compartment and the paracellular space at the basal site,
- *M*: rate constant of transport of C at the TJ,
- *P*_ap_, *P*_bl_: osmotic permeability coefficients of the apical (ap) and basal (bl) domains,
- *Q*: proportionality constant between the electrodiffusional flux of C at the TJ and the electro-osmotic water flow at that level.

### Model Variables

- *V*_cell_: cell volume,
- *C*_int_: intracellular concentration of C,
- *C*_par_: concentration of C in the paracellular space,
- *C*_ap_: concentration of C in the usl at the apical side,
- *A*_int_, *A*_par_, *A*_ap_: concentrations of A in the different compartments, which assume the same values as the corresponding concentrations of C.

### Model Equations

The system of dynamic equations is (d*x*/d*t*: time derivative of *x*):

$${\text{d}}V_{\text{cell}} /{\text{d}}t = A_{1} + A_{2} C_{\text{int}} {-}A_{3} C_{\text{ap}} + A_{4} /V_{\text{cell}} ,$$

$${\text{d}}C_{\text{int}} /{\text{d}}t = \left( {1/V_{\text{cell}} } \right)\left( {B_{1} + B_{2} C_{\text{ap}} {-}B_{3} C_{\text{int}} {-}C_{\text{int}} \left( {{\text{d}}V_{\text{cell}} /{\text{d}}t} \right)} \right),$$

$${\text{d}}C_{\text{ap}} /{\text{d}}t = C_{1} + C_{2} C_{\text{int}} + C_{3} C_{\text{par}} {-}C_{4} C_{\text{ap}} ,$$

$${\text{d}}C_{\text{par}} /{\text{d}}t = D_{1} + D_{2} C_{\text{int}} {-}D_{2} C_{\text{par}} ,$$

with

$$A_{1} = A_{\text{c}} V_{\text{w}} \left( {\left( {P_{\text{ap}} + P_{\text{bl}} } \right)\left( {C_{x} {-}S_{\text{e}} } \right) + 2P_{\text{ap}} C_{\text{e}} } \right),$$

$$A_{2} = 2A_{\text{c}} V_{\text{w}} \left( {P_{\text{ap}} + P_{\text{bl}} } \right),$$

$$A_{3} = 2A_{\text{c}} V_{\text{w}} P_{\text{ap}} ,$$

$$A_{4} = A_{\text{c}} V_{\text{w}} \left( {P_{\text{ap}} + P_{\text{bl}} } \right)M_{\text{nd}} ,$$

$$B_{1} = A_{\text{c}} K_{2} C_{\text{e}} ,$$

$$B_{2} = A_{\text{c}} K_{3} ,$$

$$B_{3} = A_{\text{c}} \left( {K_{2} + K_{3} } \right) + \left( {A_{\text{c}} + R_{\text{act}} A_{\text{c}} } \right)K_{1} ,$$

$$C_{1} = \left( {A_{\text{c}} /V_{\text{ap}} } \right)K_{\text{d}} C_{\text{e}} ,$$

$$C_{2} = \left( {A_{\text{c}} /V_{\text{ap}} } \right)K_{3} ,$$

$$C_{3} = \left( {A_{\text{tj}} /V_{\text{ap}} } \right)M,$$

$$C_{4} = \left( {A_{\text{c}} /V_{\text{ap}} } \right)\left( {K_{3} + K_{\text{d}} } \right),$$

$$D_{1} = \left( {A_{\text{par}} /V_{\text{par}} } \right)K_{4} C_{\text{e}} ,$$

$$D_{2} = \left( {A_{\text{l}} /V_{\text{par}} } \right)K_{1} ,$$

$$D_{3} = \left( {A_{\text{tj}} /V_{\text{par}} } \right)M.$$

### Osmolarities (*π*s), Fluxes of C (*J*s) and Solvent Flows (*F*s)

$$\pi_{\text{cell}} = C_{x} + \left( {M_{\text{nd}} /V_{\text{cell}} } \right) + 2C_{\text{int}} ,$$

$$\pi_{\text{ap}} = S_{\text{e}} {-}2C_{\text{e}} + 2C_{\text{ap}} ,$$

$$J_{\text{ap}} = A_{\text{c}} K_{3} \left( {C_{\text{ap}} {-}C_{\text{int}} } \right),$$

$$J_{\text{bl}} = A_{\text{c}} K_{2} \left( {C_{\text{e}} {-}C_{\text{int}} } \right){-}R_{\text{act}} A_{\text{c}} K_{1} C_{\text{int}} ,$$

$$J_{\text{l}} = A_{\text{l}} K_{1} C_{\text{int}} ,$$

$$J_{\text{ap - ext}} = A_{\text{c}} K_{\text{d}} \left( {C_{\text{ap}} {-}C_{\text{e}} } \right),$$

$$J_{\text{tj}} = A_{\text{tj}} MC_{\text{par}} ,$$

$$J_{\text{par}} = A_{\text{par}} K_{4} \left( {C_{\text{e}} {-}C_{\text{par}} } \right),$$

$$F_{\text{ap}} = A_{\text{c}} V_{\text{w}} P_{\text{ap}} \left( {\pi_{\text{ap}} {-}\pi_{\text{cell}} } \right),$$

$$F_{\text{bl}} = A_{\text{c}} V_{\text{w}} P_{\text{bl}} \left( {\pi_{\text{cell}} {-}S_{\text{e}} } \right),$$

$$F_{\text{par}} = QJ_{\text{tj}} .$$

### Numerical Values of the Parameters

V_w_ = 18 cm^3^ mole^−1^,

*S*_e_ = 3 × 10^−4^; *C*_e_ = *A*_e_ = 1.6 × 10^−4^ (mole cm^−3^),

*A*_c_ = 1.4 × 10^−6^; *A*_l_ = 10^−9^; *A*_tj_ = 1.5 × 10^−9^; *A*_par_ = 2 × 10^−7^ (cm^2^),

*R*_act_ = 0.8,

*V*_ap_ = 2 × 10^−8^; *V*_par_ = 2 × 10^−11^ (cm^3^),

*M*_nd_ = 2.2 × 10^−13^ (mole)

*C*_*x*_ = 1.4 × 10^−4^ (mol cm^−3^),

*K*_1_ = 1.8 × 10^−3^ (cm s^−1^),

*K*_3_(α) = 5.2 × 10^−6^; *K*_2_(α) = 1.2 × 10^−4^ (cm s^−1^),

*K*_3_(β) = 5.2 × 10^−6^; *K*_2_(β) = 1.2 × 10^−5^ (cm s^−1^),

*K*_d_: = 5 × 10^−4^; *K*_4_ = 3.82 × 10^−5^ (cm s^−1^),

*M* = 0.0051 (mole^−1^ cm^4^ s^−1^),

*P*_ap_ = 1.25 × 10^−2^; *P*_bl_ = 2 × 10^−2^ (cm s^−1^),

*Q* = 1.25 × 10^2^ (cm mole^−1^).

### Steady States

Depending on the numerical values assumed for the rate constants *K*_2_ and *K*_3_, there are two steady states (α and β), given by:

$$C_{\text{int}} (\upalpha ) = 1.28 \times 10^{ - 5} \;{\text{mol}}/{\text{cm}}^{3} ;\quad V_{\text{cell}} (\upalpha ) = 1.63 \times 10^{ - 9} \;{\text{cm}}^{3} ,$$

and

$$C_{\text{int}} (\upbeta ) = 1.88 \times 10^{ - 6} \;{\text{mol}}/{\text{cm}}^{3} ;\quad V_{\text{cell}} (\upbeta ) = 1.41 \times 10^{ - 9} \;{\text{cm}}^{3} .$$

For these values, we assumed that there are two cell volume thresholds, given by:

$$V_{\text{cell}} ({\text{hi}}) = 0.935V_{\text{cell}} (\upalpha ) = 1.53 \times 10^{ - 9} \;{\text{cm}}^{3} ,$$

and

$$V_{\text{cell}} ({\text{lo}}) = 1.065V_{\text{cell}} (\upbeta ) = 1.50 \times 10^{ - 9} \;{\text{cm}}^{3} .$$

These threshold values impose the characteristic cell volume dependence of the rate constants *K*_2_ and *K*_3_ assumed in this study:

$${\text{If}}\;V_{\text{cell}} > V_{\text{cell}} ({\text{hi}}),\quad K_{2} = K_{2} (\upbeta )\quad {\text{and}}\quad K_{3} = K_{3} (\upbeta ).$$

$${\text{If}}\;V_{\text{cell}} < V_{\text{cell}} ({\text{lo}}),\quad K_{2} = K_{2} (\upalpha )\quad {\text{and}}\quad K_{3} = K_{3} (\upalpha ).$$

In the intermediate range of values of *V*_cell_, the rate constants *K*_2_ and *K*_3_ retain the last values adopted in the cycle.

### Dynamic Behavior

The dynamic behavior of the system is determined by the volume dependence of the rate constants *K*_2_ and *K*_3_ assumed above. When the cell volume reaches the low threshold value *V*_cell_(lo), these rate constants assume values that tend to drive the system to steady state α, characterized by a larger value of *V*_cell_ [*V*_cell_(α)]. However, when *V*_cell_ reaches the high threshold value *V*_cell_(hi), the rate constants assume the values that drive the cell towards steady state β, characterized by a lower value of the cell volume [*V*_cell_(β)]. Successive reiterations of these modifications results in the characteristic oscillatory behavior of the model (Fig. 6). As a consequence of the periodic modifications in the concentrations of C and, consequently, in the osmolarities, the fluxes and flows also undergo periodic changes (Figs. 7, 8).

For the numerical values employed, the oscillations are characterized by a period of 9.4 s. Although the osmotic flows at both the basal and apical membranes undergo modifications between positive and negative values (Fig. 7), there is a predominance of transcellular transport in the basal to apical direction, ultimately as a consequence of net transport of C to the apical side provoked by electrodiffusion at the TJs (*J*_tj_). The numerical results obtained for the net transports of C (*J*_ap-ext_) and solvent (electro-osmotic, *F*_par_, and transcellular osmotic, *F*_bl_ + *F*_ap_) in 1 h were the following:

$$\left( {J_{\text{ap - ext}} } \right)_{{1\;{\text{h}}}} = 1.8 \times 10^{ - 6} \;{\text{mole}}\;{\text{cm}}^{ - 2} \;{\text{h}}^{ - 1} ,$$

$$\left( {F_{\text{par}} } \right)_{{1\;{\text{h}}}} = 3.73 \times 10^{ - 3} \;{\text{cm}}\;{\text{h}}^{ - 1} ,$$

$$\left( {F_{\text{bl}} + F_{\text{ap}} } \right)_{{1\;{\text{h}}}} = 1.07 \times 10^{ - 3} \;{\text{cm}}\;{\text{h}}^{ - 1} .$$

As commented in the main text, both the dynamic properties and the net fluxes and flows obtained from the numerical simulations of the proposed model compare well with experimental data.
